# Photon Devil’s staircase: photon long-range repulsive interaction in lattices of coupled resonators with Rydberg atoms

**DOI:** 10.1038/srep11510

**Published:** 2015-06-25

**Authors:** Yuanwei Zhang, Jingtao Fan, J.-Q. Liang, Jie Ma, Gang Chen, Suotang Jia, Franco Nori

**Affiliations:** 1 State Key Laboratory of Quantum Optics and Quantum Optics Devices, Institute of Laser spectroscopy, Shanxi University, Taiyuan 030006, P. R. China; 2 Institute of Theoretical Physics, Shanxi University, Taiyuan 030006, P. R. China; 3 Center for Emergent Matter Science, RIKEN, Wako-shi, Saitama 351-0198, Japan; 4 Physics Department, University of Michigan, Ann Arbor, Michigan 48109-1040, USA

## Abstract

The realization of strong coherent interactions between individual photons is a long-standing goal in science and engineering. In this report, based on recent experimental setups, we derive a strong photon long-range repulsive interaction, by controlling the van der Waals repulsive force between Cesium Rydberg atoms located inside different cavities in extended Jaynes-Cummings-Hubbard lattices. We also find novel quantum phases induced by this photon long-range repulsive interaction. For example, without photon hopping, a photon Devil’s staircase, induced by the breaking of long-range translation symmetry, can emerge. If photon hopping occurs, we predict a photon-floating solid phase, due to the motion of particle- and hole-like defects. More importantly, for a large chemical potential in the resonant case, the photon hopping can be frozen even if the hopping term exists. We call this new phase the photon-frozen solid phase. In experiments, these predicted phases could be detected by measuring the number of polaritons via resonance fluorescence.

Strong interactions between individual photons play an essential role in achieving photon quantum information processing^1^,^2^,^3^,^4^ as well as in exploring exotic many-body phenomena of light^5^,^6^,^7^. In contrast to electrons, interacting directly via Coulomb repulsion, the photon-photon interactions must be mediated by matter^8^. Being an important challenge, the realization of such matter-mediated interactions has become a long-standing goal in science and engineering. During the past decades, much theoretical^9^,^10^,^11^,^12^ and experiental^13^,^14^ effort has been made to enhance the nonlinear interaction to a strong regime at the single-photon level. Moreover, photon-photon interactions can lead to an on-site photon-blockade effect^15^,^16^, when each cavity mode interacts with a two-level atom. By further considering the novel competition between the on-site photon-blockade effect and the photon hopping in an array of coupled cavities^17^, quantum simulations^6^,^7^, based on the Jaynes-Cummings-Hubbard model^17^, have studied complex many-body phenomena in condensed-matter and atomic physics, such as the superfluid-Mott-insulator transition^18^,^19^,^20^,^21^, quantum magnetic dynamics^22^, glassy phases^23^, solid^24^,^25^ and supersolid^26^ phases, and the fractional quantum Hall effect^27^,^28^.

In this report, based on recent experimental setups, we derive a strong photon long-range repulsive interaction (PLRRI) by controlling the van der Waals force between Rydberg atoms located inside different cavities in extended Jaynes-Cummings-Hubbard lattices. We also find novel quantum phases induced by this PLRRI. For example, without photon hopping, the breaking of long-range translation symmetry induces a complex solid structure, i.e., a photon Devil’s staircase. In a “ Devil’s staircase”, any two different rational states are separated by many states. If photon hopping exists, we predict a photon-floating solid phase, due to the motion of particle- and hole-like defects. More importantly, for a large chemical potential in the resonant case, photon hopping can be frozen even if the hopping term exists. We denote this new phase the photon-frozen solid phase. In experiments, these predicted phases could be detected by measuring the number of polaritons via resonance fluorescence^29^.

## Results

### Extended Jaynes-Cummings-Hubbard model

**Figure 1:**
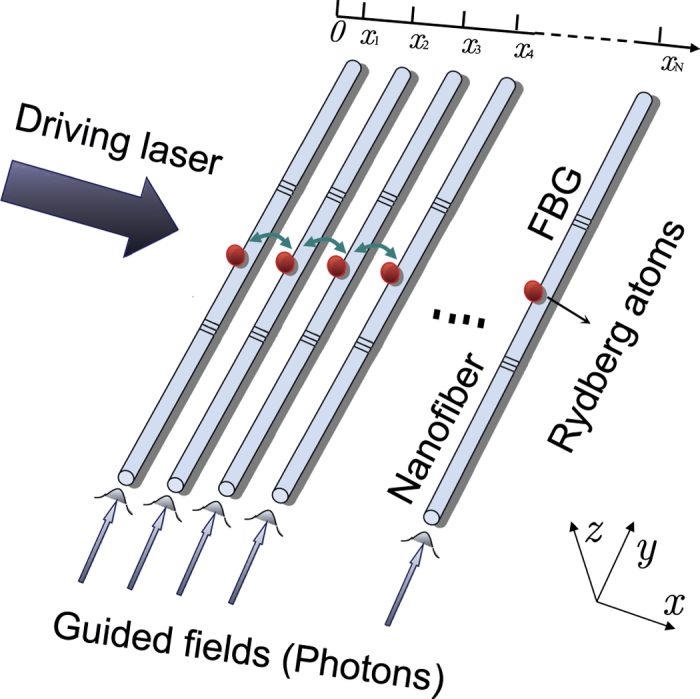
Schematic diagram of the system studied. A 1D nanofiber photonic crystal cavity array, with an ensemble of Cs Rydberg atoms (red disks) placed near each nanofiber. Photons can hop between two adjacent cavities, indicated by green double-arrows. FBG denotes the fiber Bragg grating.

**Figure 2:**
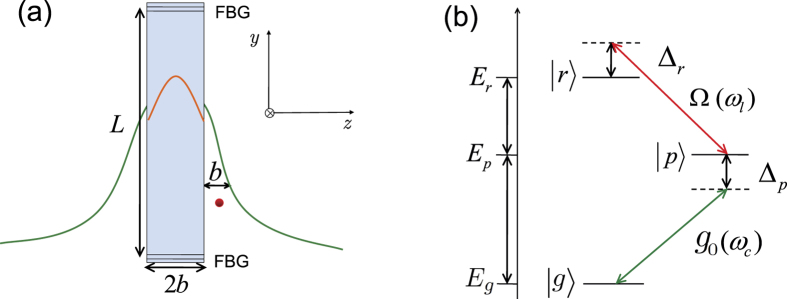
(a) The sectional plot of the *i*th atom-cavity interaction system, and (b) energy levels of a single three-level Cs Rydberg atom and their transition. In (a), the yellow and green solid curves schematically show the intensity distributions of the intracavity and evanescent fields, respectively. *b* denotes the radius of the nanofiber, which is about 0.25 *μ*m, and *L* is the length of cavity. In general, the radius *b* is smaller than the distance of the nearest-neighbor cavities, which is chosen here as *x*_*i*+1_ − *x*_*i*_ ≈ 2.4 *μ*m. In addition, FBG denotes the fiber Bragg grating. In (b), the green-arrowed line shows the photon-induced transition, whereas the red-arrowed line labels the other transition governed by the classical driving laser. The detunings are given by 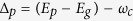 and 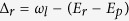, respectively.

We first propose a possible way to realize an extended Jaynes-Cummings-Hubbard model with long-range atom-atom interactions in different cavities, based on recent experimental setups^30^,^31^,^32^,^33^,^34^. As shown in Fig. 1, a series of SiO_2_ nanofibers are arranged in the same direction of a specific plane, and an ensemble of Cesium (Cs) Rydberg atoms are trapped close to each nanofiber. Each nanofiber, with radius *b* = 0.25 *μ*m, acts as a 1D photonic crystal cavity, due to its fabricated fiber Bragg-grating (FBG) structure^31^,^32^ [see Fig. 2(a)]. A guided field, whose evanescent field acts as the quantum cavity mode, propagates along the cavity *y* axis. The cavity decay rate is characterized by the parameter *κ*, which induces the photon hopping in the cavity array^35^, and the distance between nearest-neighbor cavities is about *x*_*i*+1_ − *x*_*i*_ ≈ 2.4 *μ*m. Since the evanescent field strength is sufficiently weak at the radial distance of about *b*–4*b* away from the surface of the nanofiber^36^,^37^, each adjacent nanofiber pairs located at such a distance will not lead to an efficient overlap of different cavity modes, which guarantees that the *i*th ensemble of Cs Rydberg atoms can interact only with the *i*th cavity^33^,^36^.

By using the red- and blue-detuned evanescent light fields around the optical nanofiber, a two-color optical dipole trap can be formed. This optical dipole trap should allow an ensemble of Cs Rydberg atoms to be prepared at a few hundred nanometers from the nanofiber surface^30^,^38^. For Cs Rydberg atoms, we can choose the fine-structure states 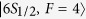 and 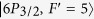 as the ground state 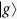 and the intermediate state 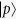, respectively, while the Rydberg state is assumed as 70*S*_1/2_. As shown in Fig. 2(b), the photon induced by the evanescent field, with wavelength 852 nm, governs the transition between the ground state 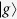 and the intermediate state 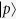, whereas the other transition between the intermediate state 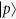 and the Rydberg state 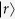 is controlled by a classical driving laser, with wavelength 510 nm, as shown in Fig. 1.

Formally, the total Hamiltonian of the system considered in Fig. 1 is


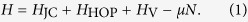


In the Hamiltonian (1), *H*_JC_ describes the interaction between the photons and the ensemble of Cs Rydberg atoms for all nanofiber photonic crystal cavities. We first consider the interaction between the photon and a single three-level Cs Rydberg atom at one cavity. In the current experimental setups^30^,^31^,^32^,^33^,^34^, the interaction between photons and the single Cs Rydberg atom is of the order of MHz (the detailed estimation will be shown in the next subsection). Therefore, in the framework of the rotating-wave approximation, the corresponding Hamiltonian is


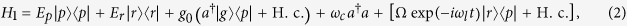


where *E*_*p*_ and *E*_*r*_ are the energies of the intermediate state 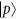 and the Rydberg state 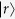, respectively, 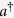 and *a* are the creation and annihilation operators of photons with frequency *ω*_*c*_, while Ω and *ω*_*l*_ are the Rabi and driving frequencies of the classical laser, respectively. When the detuning is large, we can adiabatically eliminate the intermediate state 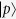, and rewrite the Hamiltonian (2) via a unitary transformation as


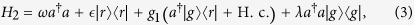


where *ω* = *ω*_*c*_*−ω*_*l*_ is the effective photon frequency, ε = *E*_*r*_ −*E*_*g*_ − *ω*_*l*_ +  Ω^2^/Δ_*p*_ is the effective transition frequency of the two-level Rydberg atom, *g*_1_ = *g*_0_Ω/Δ_*p*_ is the effective interaction strength, and λ = 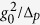. For large detuning, λ is very small and we thus can omit the interaction term 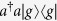.

In addition, for large detuning, *g*_1_ is also weak. In order to enhance the effective atom-photon interaction strength, here we consider an ensemble of Cs Rydberg atoms in the center of each cavity. For simplicity, we also assume that the number of Cs Rydberg atoms in each cavity is a constant *N*_*R*_. The strong van der Waals repulsive interaction between Cs Rydberg atoms in the same cavity generates a Rydberg-blocked effect, which excites only one Cs Rydberg atom^39^. In such case, we should introduce the collective ground state 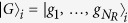, and the collective excitation state 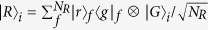.

Thus, the first term of the Hamiltonian (1) becomes


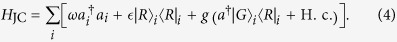


The second term in the Hamiltonian (1) governs the photon hopping between two adjacent cavities, and is


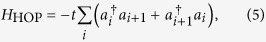


where 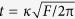 is the photon hopping rate and *F* is the cavity finesse. The third term in the Hamiltonian (1) governs the long-range van der Waals interaction between Cs Rydberg atoms in different cavities, and is


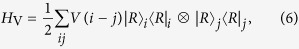


where 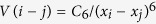 with *C*_6_ being the van der Waals coefficient, and *x*_*i*_ being the position of the *i*th cavity^40^. The long-range van der Waals interaction can induce a strong correlation between Cs Rydberg atoms in different cavities. Hereafter, we use the nearest-neighbor interaction to represent the entire van der Waals interaction, i.e., 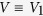, because *V*_2_ = *V*_1_/2^6^, and *V*_3_ = *V*_1_/3^6^, 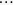. In the last term of the Hamiltonian (1), the chemical potential *μ* is the Lagrange multiplier, and the total number of polaritons is 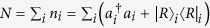.

It should be noted that a dielectric medium placed near dipoles will alter the spatial distribution of the electromagnetic field. However, for the parameters of the nanofiber and Cs Rydberg atoms considered here, this alteration can be regarded as a higher-order small quantity, compared with the direct atom-atom interaction^41^,^42^,^43^. This allows us to safely treat the interaction between Cs Rydberg atoms in different cavities as the standard long-range van der Waals force.

### Typical parameters

Before proceeding, we estimate the relevant parameters of the Hamiltonian (1) in terms of the above proposal.

- The effective photon frequency *ω* = *ω*_*c*_* − ω*_*l*_ and the effective atom transition frequency ε = *E*_*r*_ − *E*_*g*_ − *ω*_*l*_ +  Ω^2^*/*Δ_*p*_. These two parameters can be well controlled by the driving frequency *ω*_*l*_ of the classical laser. Thus, these can have suitable values as required experimentally.
- The collective atom-photon interaction strength 
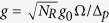
. In our considered nanofiber photonic crystal cavity, 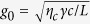, where *η*_*c*_ is the channeling efficiency, *c* is the light velocity, *L* is the cavity length^44^,^45^. It should be noted that since the Cs Rydberg atoms considered here are tightly trapped, the decay *γ* of the Rydberg superatom is enhanced^46^ by *γ* = *N*_*R*_Γ, where Γ is the decay of an isolated Cs Rydberg atom in the state 70*S*_1/2_, due to the supperradiant effect^47^. The Rabi frequency and the detuning are chosen here as Ω/2π ~ 100 MHz and Δ_*p*_/2π ~ 1 GHz, respectively, which fulfill the adiabatic elimination condition, 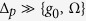. In addition, for the two-color optical dipole trap, with wavelengths^33^ 1064 nm and 780 nm, respectively, the number of Cs Rydberg atoms of each ensemble can be of the order of 10^4^. Therefore, the collective atom-photon interaction strength reaches 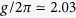 GHz, when *η*_c_*/*2π = 0.01 (see Ref. 33), *γ* = 27.5 MHz (Γ/2π = 0.55 kHz), *L* = 10 mm, and *N*_*R*_ = 5 × 10^4^. If the atomic number density is increased, this collective atom-photon interaction strength *g* can increase rapidly, because it is proportional to 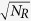.
- The van der Waals interaction strength *V*(*i* − *j*) = *C*_6_/(*x*_*i*_ − *x*_*j*_)^6^. Based on the aforementioned energy level structures^48^,^49^, the van der Waals coefficient is *C*_6_ ≈ 610 GHz·*μ*m^6^. For the distance *x*_*i*+1_ − *x*_*i*_ ≈ 2.4 *μ*m, the interaction strength between the nearest-neighbor sites is *V*_1_/2π ≈ 500 MHz, i.e., *V*/2π = *V*_1_/2π ≈ 500 MHz. This interaction strength can be modified by changing the distance of the nearest-neighbor cavities.
- The cavity decay rate * κ* and the photon hopping rate *t*. In the nanofiber photonic crystal cavity considered in Fig. 2(a)^44^,^45^, *κ* = *πc*/*FL*. In current experimental setups^34^, *F *≈ 500. Thus, *κ*/2π = 30 MHz and *t*/2π = 628 MHz, when *L* = 10 mm. Both the cavity decay rate and the photon hopping rate can be controlled by changing the cavity length.

The above parameters show two basic features: 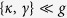 and *V* = *V*_1_ ~ *g*. The condition 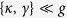  implies that we may safely neglect the influence of the decay of both cavity and atom, because these only change slightly the phase boundaries^50^,^51^. In addition, using the above parameters, we also estimate that the atomic number density of each cavity is of the order of 10^12^ cm^−3^. For such a typical density, the dephasing time of the collective states 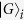 and 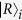 which are induced by the atomic collision, can, at least, reach the order of microseconds. This is much larger than the time scales of *κ*^−1^ and *g*^−1^, and can thus be neglected^48^,^52^. This guarantees the validity of our effective two-level model in Eq. (4)^39^,^52^.

### Photon long-range repulsive interaction

We now construct a strong PLRRI in terms of the Hamiltonian *H*_V_. We begin to address the simplest case, *κ* = *V* = 0, in which the Hamiltonian (2) reduces to


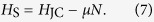


The eigenstates of the Hamiltonian *H*_S_ are given by


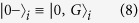


for *n* = 0, and


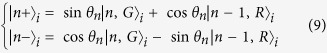


for *n* ≥ 1, where 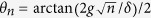 and *δ* =  *ω* − *ε* is the detuning. The corresponding eigenvalues are *E*_0_ = 0 and


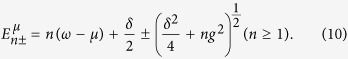


Since here we investigate the lower-energy behavior, only the lower polariton branch 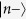 is considered^17^. Thus, the Hamiltonian *H*_S_ is rewritten as


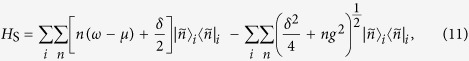


where 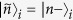. The second term of the Hamiltonian *H*_S_ leads to an even distribution of polaritons, which provides an effective on-site repulsive interaction between photons^17^. When 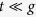 the rotating-wave approximation is reasonable, and thus the hopping term becomes


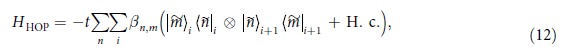


where 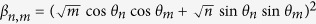 and 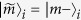, with *m* = *n* + 1. In addition, since the upper polariton branch 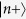 has the higher probability of Rydberg excitation (stronger repulsive interaction), we also only consider the projection of the van der Waals interaction into the lower polariton branch 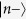. Thus, the corresponding Hamiltonian becomes


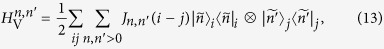


where


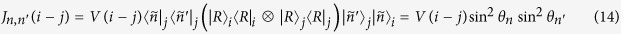


is the effective interaction strength. Since *V*(*i* − *j*) > 0, and moreover, *V* = *V*_1_ ~ *g*, Eq. (13) demonstrates explicitly that the van der Waals interaction generates a strong PLRRI. As will be shown below, this strong PLRRI leads to non-trivial quantum phases exhibiting photon solid states.

### Quantum phases

We investigate quantum phases and phase diagrams by perturbation theory and a mapping into an effective Hamiltonian. For instance, when the chemical potential *μ* is weak, the high-occupancy-photon states (*n* > 1) of the Hamiltonian (2) are not considered. In such case, we rewrite the Hamiltonian (2) in a reduced Hilbert space, with *n* = 0, 1, as


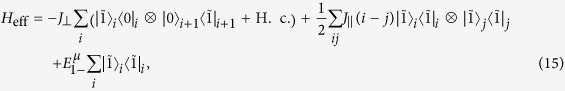


where 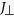 = *t*cos^2^*θ*_1_, 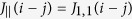 and 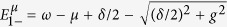 is the single-particle energy of the 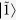 state. This effective photon hopping rate 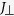 can be easily tuned by the detuning *δ*, since *θ*_1_ = arctan(2*g*/*δ*)/2. In addition, for the low-energy effective Hamiltonian (15), it is convenient to introduce a renormalized nearest-neighbor van der Waals interaction 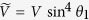 to simplify the discussions about phase diagrams, as shown below.

**Figure 3:**
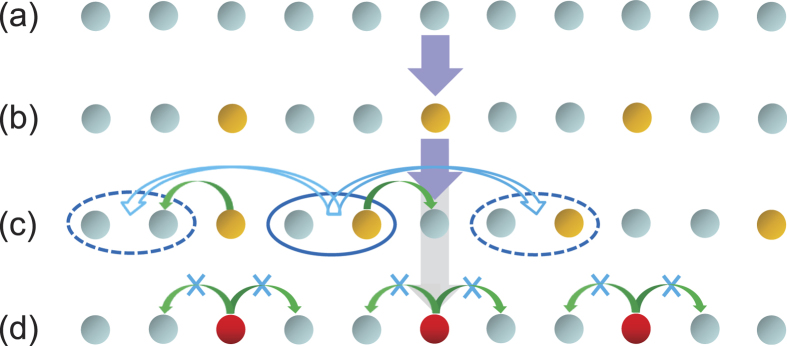
Photon distributions of each cavity for different effective strengths *V* of the van der Waals interaction, when increasing the chemical potential *μ*. (a–b) *t* = 0 with a weak *V*, (c) *t* ≠ 0 with a weak *V*, and (d) *t* ≠ 0 with a large *μ* and a strong *V*. The vacuum state 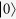 is denoted by light blue disks, and the photon excitation state 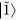 is shown in orange. (a) In the initial state, every cavity is in its vacuum state. When increasing *μ*, cavities can be excited. Due to existence of the PLRRI, the 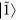 states are generated one by one and deviated from each other. Thus, the ground states of system are a series of photon solid phases, with different fraction filling factors (from low to high). We call it photon Devil’s stair case. As an example, (b) shows a photon solid phase with a period of 3 sites 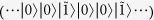. (c) Melting of this photon solid phase. A particle-like defect with the unit cell 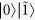 is shown inside the blue solid elliptic curve in (c). When a photon on the edge of the defect hops one site, this defect will move three sites (the new possible positions are labeled by dashed ellipses). (d) Plot of a photon-frozen solid phase, which is composed of the 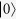
and 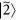 (red color) states.

We first consider the case without photon hopping (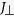 = 0). At the initial time, we assume that every cavity is in its vacuum state, as shown in Fig. 3(a). When increasing the chemical potential *μ*, photons in some cavities can be excited, due to the existence of the PLRRI (without the PLRRI, all cavities are excited identically^17^), and some 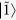 states emerges, as shown in Fig. 3(b). The corresponding critical point is


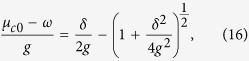


derived from 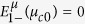. Since the 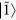 states are generated one by one and deviate from each other, the system exhibits photon solid states, which are mainly governed by different filling factors


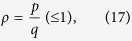


with *p* and *q* being both integers. In order to quantitatively determine the filling factor *ρ*, we introduce 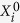 and 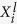, where 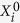 is the position of the *i*th 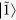 state and 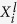 is the distance to the *l*th next 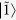 state, satisfying 
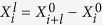
. When the ground-state energy is minimized for all sites, we have


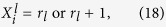


where *r*_*l*_ < *l*/*ρ* < *r*_*l*_ + 1, and satisfy the relation^53^,^54^


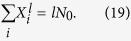


In Eq. (19), *N*_0_ is the total number of cavities. For a given filling state, the repulsive interaction energy of the 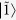 states can be estimated by applying the relations in Eqs. (18)–(19), 74 to the Hamiltonian (15). Moreover, the corresponding phases are stable if it costs energy to add or remove a particle and rearrange the structure.

### Photon solid phase

We define the photon solid phase, with the filling factor *ρ*, as 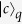. If we add one 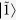 state, 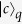 becomes 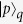 and the 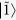 states are crowded. To minimize the repulsive energy, the summation of distances between the 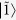 states must be a minimum. Thus, the most likely rearrangement structure is that some pairs of the adjacent 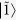 states are shortened by one site^53^,^55^. By considering the periodic boundary condition and relations in Eqs. (18)–(19), 74, *r*_*l*_ 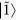 state pairs with 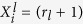 must be replaced by (*r*_*l*_ + 1) 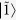 state pairs with 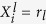. In addition, at the phase-transition point, there is no energy gap^55^ between 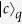 and 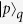, i.e., 
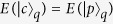
, and the critical point is thus obtained by


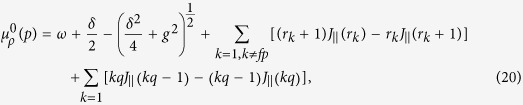


where *f* is any integer (see Methods section). Similarly, if we remove one 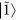 state, 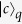 turns into 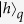, and the corresponding critical point is given by (see Methods section)


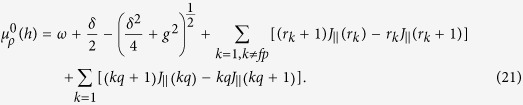


In terms of the obtained 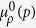 and 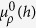, the stability interval, 
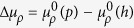
, is evaluated as


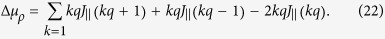


The expression for Δ*μρ* shows that the stability interval is only dependent on *q*, and moreover, decreases rapidly when increasing *q*. This means that the photon solid phases with *p* = 1, i.e., *ρ* = 1/*q* = 1/2, 1/3, 1/4,…, are more likely to be observed. Below, we mainly address these phases.

### Photon Devil’s staircase

**Figure 4:**
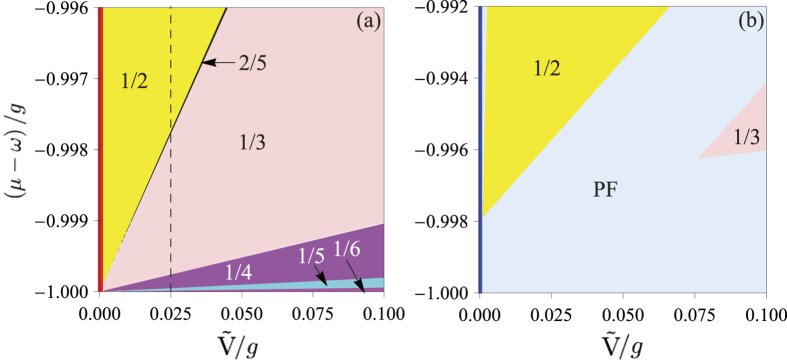
The filling factor *ρ* = *p*/*q* as a function of the chemical potential *μ* and the renormalized effective strength 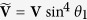 of the van der Waals interaction, when (a) *J*_⊥_/*g* = 0 and (b) *J*_⊥_/*g* = 0.001. In (a), the ground states of system are the photon solid phases. For finite 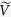, when increasing *μ*, excitation of the cavities is favorable, and *ρ* varies “jumpily” from 1/6, 1/5, 1/2, 1/4, 1/3, 2/5, to 1/2. This behavior clearly shows a devil’s staircase. On the contrary, when increasing 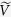 for a finite *μ*, the PLRRI prevents excitation of the cavities, and *ρ* decreases “jumpily” from 1/2 to 1/6. In (b), when the photon hopping exists, the photon solid phases melt, attributed to the motion of particle- and hole-like defects. Thus, the photon-floating solid phase (PF) emerges.

In Fig. 4(a), we plot the filling factor *ρ* as a function of the chemical potential *μ* and the renormalized effective strength 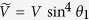 of the van der Waals interaction, in terms of the obtained 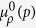 and 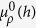 in Eqs. (20) and (21). For 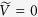, *ρ* = 1, as expected [see the red solid line in Fig. 4(a)]. However, the results for finite 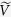 [for example, 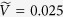 g; see the black dashed line in Fig. 4(a)] are very interesting. When increasing *μ*, *ρ* is not a constant, but varies “jumpily” from 1/6, 1/5, 1/2, 1/4, 1/3, 2/5, to 1/2. The reason is that when increasing *μ*, 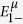 decreases, and excitation of the cavities is thus favorable. This behavior clearly shows a Devil’s staircase^55^,^56^. Moreover, this Devil’s staircase could be detected experimentally by measuring the mean-photon number 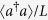, since 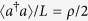, and thus here called the *photon Devil’s staircase*. However, when increasing 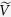, *ρ* varies jumpily from high to low because the PLRRI prevents the photon excitation.

Recently, the photon nearest-neighbor interaction was studied and a photon solid state was predicted^24^. In that case, the *Z*_2_ symmetry, translated by one site, has been broken. Here the PLRRL generates a long-range translation symmetry, whose breaking induces the photon Devil’s staircase. Moreover, it leads to other non-trivial phases when the photon hopping exists.

Notice that between the adjacent photon solid phases, with *ρ* = 1/*q* and 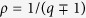, respectively, there are many transition states which have different numbers of defects. Here we define the pairs of the 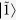 states with shorter (longer) distance as a particle- (hole-) like defect structure. Since these states have very small stability intervals, they should be hard to observe when 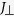 = 0, and thus not plotted in Fig. 3(b). However, when 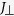 ≠ 0, they play an important role for the ground-state properties, because of the motion of the defects, as shown in Fig. 3(c). Especially, when the hopping energy is negative, the states with defects may be more stable than the adjacent photon solid states. Thus, the photon solid phases melt and a photon-floating solid phase^57^ can emerge. In general, it is difficult to fully characterize this process. However, in the region close to the phase-transition point, the repulsive interaction between the defects only allow one defect. Thus, the phase boundary can be estimated by comparing the energy of the photon solid state 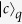 with that of the state with one defect. Using a perturbative method, we obtain the following phase boundaries (see Methods section):


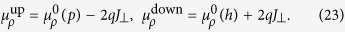


Equation (23) shows that the hopping energies of the defects reduce the regions where the photon solid phases exist, because 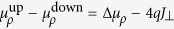. In particular, when 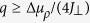, 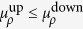 , and thus the energy bands of the particle- and hole-like defect states cross and the photon solid phases cannot exist. This is the reason why only the photon solid phases, with *ρ* = 1/2 and *ρ* = 1/3, can emerge in Fig. 4(b). From Fig. 4(b), we also see that the regions where the photon solid phases exist are very small, and are melted for a smaller 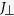 (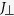/*g* = 0.001). This implies that the hopping term can be treated as a perturbation. So the results from the phase boundaries in Eq. (23) are reasonable. Strictly speaking, in the photon-floating solid phase, the total number of the 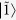 states is sensitive to the fluctuation of the parameters, and also *ρ* and 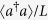 are hard to calculate in that phase. Recently, the quantum Monte Carlo method has been used to solve this problem^58^. When 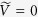, the photon-floating solid phase disappears [see the blue line in Fig. 4(b)].

### Photon-frozen solid phase

**Figure 5:**
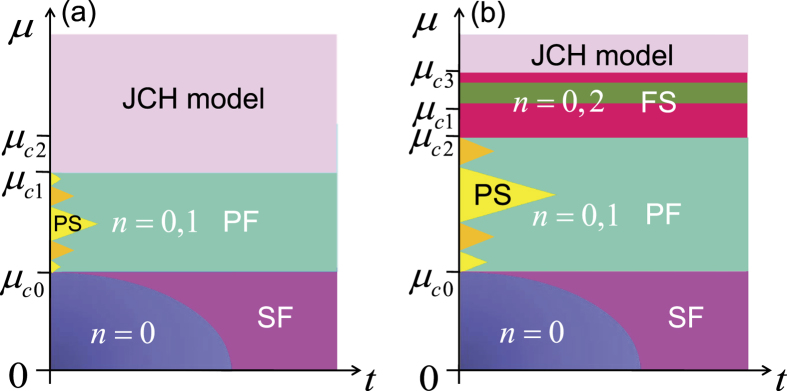
Schematics of the ground-state phase diagrams as functions of the chemical potential *μ* and the photon hopping rate *t*, when *δ* = 0. In (a) the PLRRI is weak and all cavities are excited to the 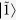 states before the higher-photon-occupancy states emerge. This can be determined by considering *μ*_*c*1_ < *μ*_*c*2_. In (b), the PLRRI is strong and the photon-frozen solid phase occurs. This can be determined by considering *μ*_*c*1_ > *μ*_*c*2_. When *μ* > *μ*_*c*1_ and *μ* > *μ*_*c*3_, all cavities in (a) and (b) are excited identically, respectively. Here, SF, PS, PF, and FS denote the following phases: superfluid, photon solid, photon-floating solid, and photon-frozen solid, respectively. JCH stands for Jaynes-Cummings-Hubbard. This figure is not to scale.

Finally, we address the case of a strong chemical potential *μ*, in which the higher-photon-occupancy states in some cavities can occur, and moreover, the single-particle energy of the 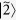 state, 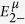, is close to that of the 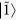 state, 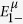, (here we omit the case *n* > 2). In this case, there are three kinds of repulsive interactions: between the 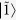 and 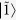 states, between the 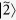 and 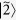 states, and between the 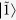 and 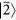 states. Moreover, the photon hopping has two channels, from the 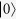 to 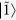 states and from the 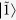 to 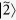 states. These two channels are very complex. However, in the resonant case (*δ* = 0), sin^2^*θ*_*n*_ = 1/2, and 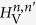 is thus independent of *n*. This indicates that the photon numbers of the excited cavities are only determined by 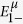 and 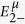. When the PLRRI is not sufficiently strong, the lattice can be fully filled in the weak-*μ* region. In this region, 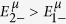, and the ground state, still governed by the Hamiltonian (15), is thus composed of the 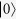 and 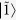 states. By increasing *μ*, *ρ* increases from 0 and reaches 1. Further increasing *μ*, all cavities can be excited with uniform photon numbers, which is similar to that of the standard Jaynes-Cummings-Hubbard model, as shown in Fig. 5(a).

However, there is a non-trivial case for a strong PLRRI, as shown in Fig. 5(b). In such case, the photon solid phases can exist in the strong-*μ* region. But we cannot ensure that the lattice is fully filled by the 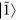 states, due to inversion of 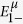 and 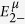. This process can be determined by comparing *μ*_*c*1_ ≈ ω − *g* + 1.0175 *V*, obtained by making *ρ* = 1 in 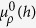, with the other critical point *μ*_*c*2_ ≈ ω + 0.414 *g* (the degenerate point of 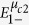 and 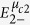). When *V* > 0.576 *g*, *μ*_*c*1_ > *μ*_*c*2_, and there is a transition from the 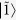 to 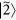 states in the excited cavities. Thus, this transition induces a new crystalline configuration, which is composed of the 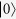 and 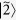 states. The corresponding low-energy behavior is governed by a new effective Hamiltonian


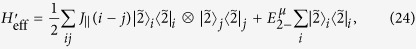


where 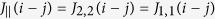, and 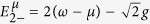. Since


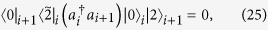


the photon hopping is always frozen even if *t* exists. We denote the corresponding phase as the *photon-frozen solid phase*. In this phase, the fractional filling structure of the 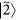 states is robust, i.e., it is not easily destroyed by the photon hopping. In terms of the Hamiltonian (24), when further increasing *μ;* to satisfy 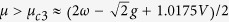, the lattice can be fully filled by the 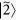 states, as shown in Fig. 5(b)

## Discussion

In summary, we have achieved a strong PLRRI by controlling the van der Waals interaction of Rydberg atoms located in different cavities in extended Jaynes-Cummings-Hubbard lattices, and then predicted novel quantum phases. Since the atom-cavity polariton can be easily controlled experimentally^59^,^60^, our proposal offers a new way to control the interaction between individual photons. In addition, our proposal might help to explore rich many-body phenomena of light and quantum nonlinear optics, as well as potential applications to quantum information and computing.

## Methods

### Derivation of Eqs. (20) and (21)

We have described the low-energy behavior of the Hamiltonian (1) by an effective Hamiltonian (15). Moreover, we have also pointed out that when 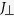 = 0, there is a succession of photon crystal states with different filling factors, denoted as a photon Devil’s staircase structure, and the energy gap of the photon crystal states can be calculated in terms of Eqs. (18) and (19), i.e., 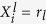 or *r*_*l*_ + 1, and 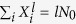. For example, we define the crystalline ground state, with the filling factor *ρ* = *p*/*q*, as 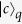. By adding one 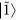 state, the crystalline ground state 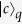 becomes 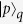. After rearranging the 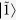 states, the distance *r*_*l*_ between the 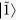 states is changed. Using Eqs. (18) and (19), *r*_*l*_ 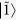 state pairs with 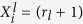 must be replaced by (*r*_*l*_ + 1) 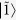 state pairs with 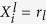. So the corresponding energy shift, 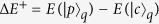, is calculated as


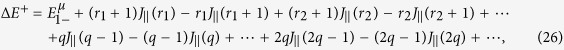


where *r*_*p*_ = *q*, *r*_2*p*_ = 2*q*,…, have been inserted^55^. Similarly, by removing one 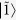 state from 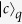, we obtain a new state 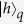. The corresponding energy shift, , 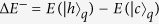
is calculated as


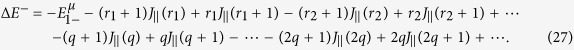


These equations govern the energy gap of the photon crystal state 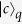. Obviously, at the phase-transition point, the energy gap is closed, i.e., Δ*E*^±^ = 0. Using the expression 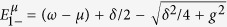, we can derive the critical point of the chemical potential. The critical point between 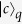 and 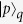 is


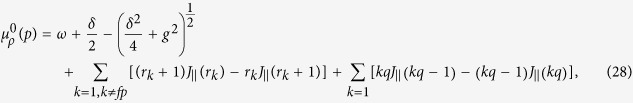


where *f* is any integer. Similarly, the critical point between 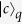 and 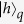 is given by


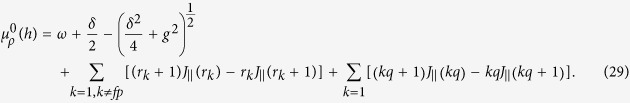


### Derivation of Eq. (5)

We define


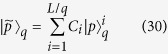


as a state with a one particle-like defect, where the index *i* denotes the position of the defect and *C*_*i*_ is its coefficient. For simplicity, we only consider the lowest order of the photon hopping: the motion of the defect. Inserting 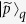 into equation 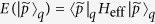, we obtain


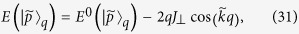


where 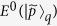 is the summation of the on-site and repulsive energies, 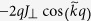 is the hopping energy band of a defect with wave number 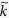. The phase boundary is determined by the lowest energy of 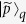, i.e., 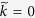 and 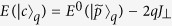. Thus, the upper bounds of the photon solid phases are given by


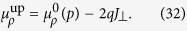


Similar to the above discussions, the lower bounds of the photon solid phases are obtained by


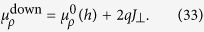


## Additional Information

**How to cite this article**: Zhang, Y. *et al.* Photon Devil’s staircase: photon long-range repulsive interaction in lattices of coupled resonators with Rydberg atoms. *Sci. Rep.* **5**, 11510; doi: 10.1038/srep11510 (2015).
